# Determination of vitamin K encapsulated into lipid nanocarriers by dispersive liquid–liquid microextraction combined with liquid chromatography–atmospheric pressure chemical ionization–tandem mass spectrometry

**DOI:** 10.1002/fsn3.3104

**Published:** 2022-10-17

**Authors:** Guijae Yoo, Tae‐Eun Kim, Min Hyeock Lee, Bum‐Keun Kim, Hae Won Jang

**Affiliations:** ^1^ Research Group of Food Processing Korea Food Research Institute Wanju Korea; ^2^ Department of Food Science and Biotechnology Kyung Hee University Yongin Korea; ^3^ Department of Food Science and Biotechnology Sungshin Women's University Seoul Korea

**Keywords:** dispersive liquid–liquid microextraction, liquid chromatography–tandem mass spectrometry, response surface model, vitamin K

## Abstract

Dispersive liquid–liquid microextraction was used in conjunction with liquid chromatography–atmospheric pressure chemical ionization–tandem mass spectrometry to quantitate vitamins K1 and K2 in vitamin‐fortified emulsions, and vital microextraction parameters were optimized using response surface methodology coupled with Box–Behnken design. Under optimal microextraction conditions, highly linear (*R* ^2^ > .999) calibration curves were obtained for both vitamins in a broad concentration range (1–1000 μg/L), and vitamin recoveries exceeded 90%. The detection and quantitation limits equaled 1.89 and 5.72 μg/L for vitamin K1, respectively, and 5.00 and 15.15 μg/L for vitamin K2, respectively. When applied to vitamin‐K‐loaded nanoemulsions and solid lipid nanoparticles, the developed method achieved excellent results, outperforming the currently employed Korean Food Code method, and therefore holding great promise for the quantitation of vitamin K in vitamin‐fortified food products.

## Graphical abstract

The proposed method uses dispersive liquid–liquid microextraction (DLLME) in conjunction with liquid chromatography–atmospheric pressure chemical ionization–tandem mass spectrometry (LC‐APCI‐MS/MS) and is optimized using the response surface model (RSM) based on a Box–Behnken design (BBD). The developed method is powerful and reliable, and is a suitable alternative to the current Korean Food Code analytical method.

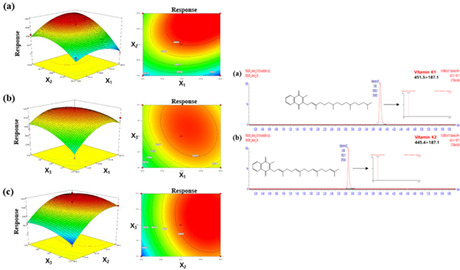

## 1 INTRODUCTION

Vitamin K is a lipid‐soluble vitamin found in two natural forms, viz., phylloquinone (vitamin K1) and menaquinone (MK; vitamin K2) (Harshman & Shea, 2016), which have the same 2‐methyl‐1,4‐naphthoquinone (menadione) core but differ in the length and saturation degree of the isoprenoid tail attached to the core at position 3 (Fu et al., 2017). Vitamin K1 is present in all photosynthetic plants, where it acts as an essential photosystem I electron carrier (Gross et al., 2006); therefore, the best sources of this vitamin are dark green leafy vegetables, such as mustard greens, turnip greens, romaine lettuce, and chicory (Al Rajabi et al., 2012). In contrast, vitamin K2 is predominantly of microbial origin and exists in different forms that are referred to as MK‐4 to MK‐13 according to the number of isoprenyl units in the isoprenoid tail (Fernandez & Collins, 1987). Both vitamin K1 and menadione can be converted into MK‐4 in animal tissue (Suttie, 1995).

An ideal sample preparation technique for food analysis applications should remove interfering substances as completely as possible and be compatible with a wide range of sample matrices (Vickers, 2017). Although traditional extraction methods, such as liquid–liquid extraction and solid‐phase extraction, are widely used for sample pretreatment (Titato & Lanças, 2005), they exhibit certain drawbacks, which has inspired the development of alternative pretreatment methods like microextraction (Ibrahim et al., 2017; Kim et al., 2022).

In particular, dispersive liquid–liquid microextraction (DLLME, first reported in 2006; Rezaee et al., 2006) offers the advantages of high accuracy, low solvent consumption, short extraction time, and high enrichment factors (Viñas et al., 2014) and has been successfully used to extract highly and moderately lipophilic compounds like aromatic amines, polycyclic aromatic hydrocarbons, and pesticide residues (Andraščíková et al., 2013; Galuch et al., 2019; Liu et al., 2014; Özkan et al., 2019). In DLLME, the analyte is transferred from the aqueous phase (sample solution) to the organic phase (extraction solvent) with the help of a dispersive solvent, which reduces the polarity of the aqueous phase and increases the solubility of the target analyte in the organic phase (Farajzadeh et al., 2016; Lee et al., 2018, 2019).

Most vitamin K analysis methods reported to date rely on high‐performance liquid chromatography (HPLC) with different types of detection (e.g., ultraviolet, fluorescence, or electrochemical detection) after post‐column derivatization (Dabre et al., 2011; Marinova et al., 2011). Among the detection techniques, fluorescence detection is used most commonly but is generally less sensitive than mass‐spectrometric detection (Jäpelt & Jakobsen, 2016).

Herein, DLLME coupled with liquid chromatography–atmospheric‐pressure chemical ionization–tandem mass spectrometry (LC‐APCI‐MS/MS) was tested as a method of vitamin K quantitation in vitamin‐fortified foods, and vital DLLME parameters, such as NaCl content and extraction and dispersive solvent volumes, were optimized using response surface methodology coupled with Box–Behnken design. The optimized procedure was validated in terms of linearity, repeatability, accuracy, limit of detection (LOD), and limit of quantitation (LOQ) and used to quantitate vitamin K in correspondingly loaded lipid nanocarriers.

## 2 MATERIALS AND METHODS

### 2.1 Chemicals and reagents

Analytical standards of vitamins K1 and K2 were obtained from Sigma‐Aldrich. Standard stock solutions (1 mg/L) were prepared in methanol and stored in amber glass vials at −20°C. Each standard solution was diluted with methanol to obtain seven aqueous working solutions (1, 5, 10, 50, 100, 500, and 1000 μg/L) that were used to construct standard calibration curves. HPLC‐grade methanol, ethanol, acetonitrile, carbon tetrachloride, chloroform, and water were purchased from J.T. Baker. Carbon disulfide and acetone were purchased from Sigma‐Aldrich.

### 2.2 Sample preparation

Two types of vitamin‐K‐fortified samples (nanoemulsions [NEs] and solid lipid nanoparticles [SLNs]) were prepared. NEs were prepared by dissolving vitamin K (500 mg), lecithin (25 g), TW80 (25 g), and olive oil (50 g) in distilled water (900 ml). SLNs were prepared by dissolving vitamin K (250 mg), lecithin (2.5 g), TW80 (2.5 g), and palm oil (5 g) in water (90 ml) at 65°C for 5 min with subsequent rapid dispersion in an ice bath upon stirring at 90 *g* for 10 min using a Ultra Turrax (IKA Staufen GmbH, Germany) equipped with 18 G stirring probe. To reduce the matrix effect, the fortified samples were diluted 10,000‐fold with purified water to a final concentration of 50 μg/L prior to analysis.

### 2.3 DLLME procedure

The extraction solvent (chloroform, 300 μl) was mixed with the dispersive solvent (methanol, 1 ml), and the mixture was rapidly injected into a conical glass tube containing a diluted sample (4 ml) and shaken for several seconds. The rapid injection of the solvent mixture produced a cloudy solution containing microdroplets of the extraction solvent dispersed in the aqueous phase (Lee et al., 2019). The sample was subsequently centrifuged for 5 min at 1500 *g*, and the bottom phase (sedimented extraction phase microparticles) was collected and evaporated to dryness in a flow of nitrogen. The residue was reconstituted with acetonitrile (0.5 ml) and transferred to a vial using a syringe for analysis by LC‐APCI‐MS/MS.

### 2.4 Experimental design and optimization by response surface methodology

The extraction and dispersive solvents were individually optimized before the application of the response surface model (RSM). Carbon tetrachloride, chloroform, dichloromethane, and carbon disulfide were tested as extraction solvents, while acetonitrile, methanol, and acetone were tested as dispersive solvents. After selecting the best dispersive and extraction solvents, we used Box–Behnken design (BBD) to construct a second‐order RSM. The extraction solvent volume (*X*_1_), dispersive solvent volume (*X*_2_), and NaCl content (*X*_3_) were selected as independent factors and studied at three levels (Table 1). The BBD included 15 experiments with three center points. The chromatographic peak area of vitamin K1 was used to evaluate extraction efficiency. The experimental data were fitted to a second‐order polynomial model, as shown in Equation (1).

(1) $$Y=\beta_{0}+\sum_{i=1}^{k}\beta_{i}X_{i}+\sum_{i=1}^{k}\beta_{\mathrm{ii}}{X_{i}}^{2}+\sum_{i=1}^{k-1}\sum_{j=2}^{k}\beta_{\mathrm{ij}}X_{i}X_{j},$$

where *X*_*i*_ and *X*_*j*_ are independent variables influencing response *Y*; *β*_0_, *β*_*i*_, *β*_*ii*_, and *β*_*ij*_ are the regression coefficients for the intercept, linear, quadratic, and interaction terms, respectively; and *k* is the number of variables (Liyana‐Pathirana & Shahidi, 2005). Design‐Expert Software version 7.0.0 (Stat‐Ease) was used for experimental design construction and data analysis.

**TABLE 1:** Experimental parameter ranges and variable levels used for Box–Behnken design

Variable	Factor	Level		
	*X*_*i*_	Low (−1)	Middle (0)	High (+1)
Dispersive solvent volume (μl)	*X*_1_	500	1750	3000
Extraction solvent volume (μl)	*X*_2_	100	300	500
NaCl content (%)	*X*_3_	0	6	12

### 2.5 LC‐APCI‐MS/MS analysis

Chromatographic separation was performed using an ACQUITY UPLC system, coupled to a Xevo triple‐quadrupole mass spectrometer equipped with an APCI source (Waters). An ACQUITY UPLC® BEH C_18_ column (130 Å, 2.1 × 150 mm i.d., particle size = 1.7 μm) was used for analysis, and 90:10 (v/v) acetonitrile: water (solvent A) and methanol (solvent B) were used as eluents. The elution program was as follows: 0–0.5 min, 100% A; 2.5–4.5 min, 0% A; 5–6 min, 100% A. The flow rate equaled 0.5 ml/min, and the total runtime was 6 min. Vitamin K was determined in multiple reaction monitoring (MRM) mode at a capillary voltage of 1.0 kV, a cone voltage of 20 V, and extractor voltage of 3.0 V, a source temperature of 130°C, and a desolvation temperature of 450°C using nitrogen as a collision gas. Masslynx™ version 4.1 software (Micromass, Waters) was used for instrumental control, data acquisition, and processing. The MRM parameters, retention times, and collision energies used for vitamin K analysis are listed in Table 2.

**TABLE 2:** MRM parameters used for vitamin K analysis

Analyte	Retention time (min)	Precursor ion (*m*/*z*)	Collision energy (eV)	Product ion (*m*/*z*)
Vitamin K1	3.80	451.5	35	187.1
Vitamin K2	2.88	445.4	25	187.1

### 2.6 Method validation

Diluted solutions of vitamin K standards were injected in triplicate, and calibration curves were constructed by plotting analyte peak area versus concentration. The LOD was measured as the concentration affording the weakest detectable peak with a signal to noise (S/N) ratio of 3, and the LOQ was determined as the lowest concentration allowing quantitative analysis and affording a peak with S/N = 10 (ICH, 2005). Precision was assessed by the intra‐day and inter‐day precisions and expressed as the relative standard deviation (RSD). Three replicate measurements were performed at three concentrations (5, 10 and 50 μg/L) using standard solutions. Recoveries were evaluated as the ratio of the amounts of extracted analytes and initial analytes from a blank sample.

## 3 RESULTS AND DISCUSSION

### 3.1 Selection of extraction and dispersive solvents

**FIGURE 1:**
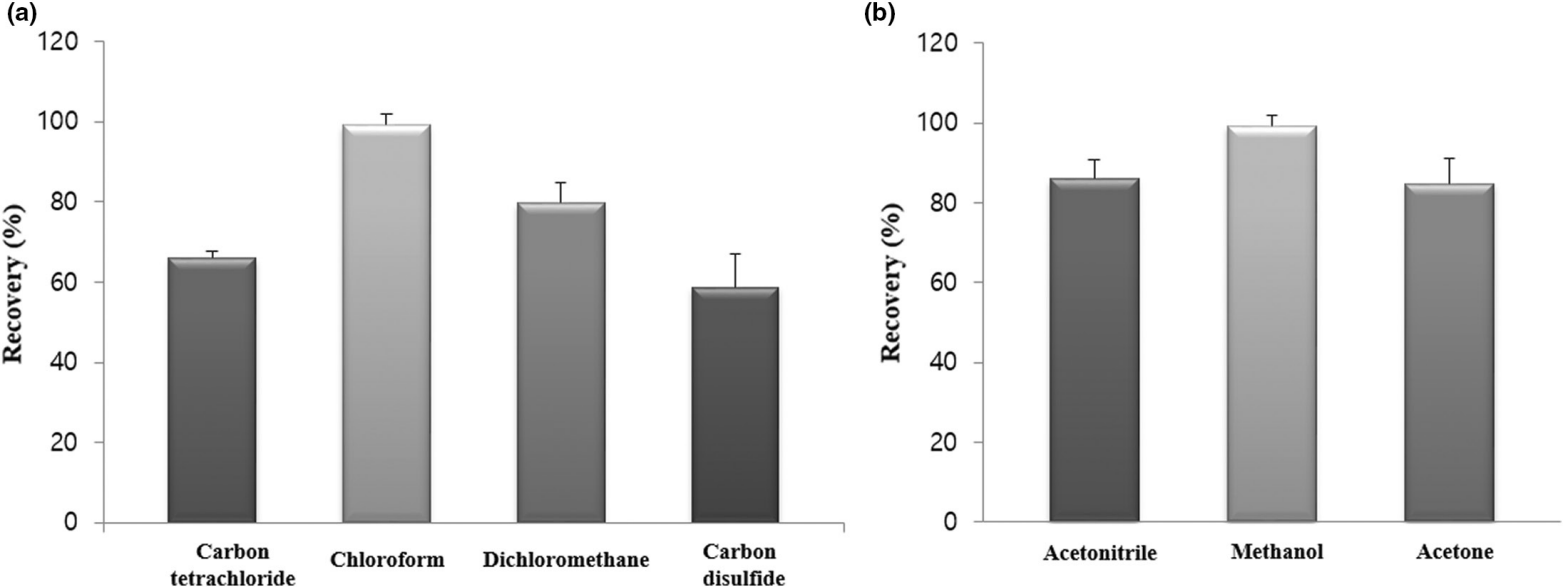
Results of (a) extraction and (b) dispersive solvent optimization.

The performances of extraction and dispersive solvents were assessed in terms of analyte recovery and extraction efficiency, respectively. As a result, chloroform and methanol were selected as optimal extraction and dispersive solvents, that is, as those affording the highest recovery and extraction efficiency (Figure 1).

### 3.2 RSM‐based optimization of DLLME procedure

Vital DLLME parameters, viz., extraction solvent volume (*X*_1_), dispersive solvent volume (*X*_2_), and NaCl content (*X*_3_), were optimized using response surface methodology coupled with Box–Behnken design (Rais et al., 2021). The relationship between these parameters and vitamin K content was determined as

(2) $$\text{Vitamin}\;K\;\text{content}=97.77+10.25X_{1}+33.99X_{2}+8.89X_{3}+15.76X_{1}X_{2}-7.81X_{1}X_{3}-3.46X_{2}X_{3}-21.02X_{1}^{2}-25.66X_{2}^{2}-12.64X_{3}^{2}.$$

**FIGURE 2:**
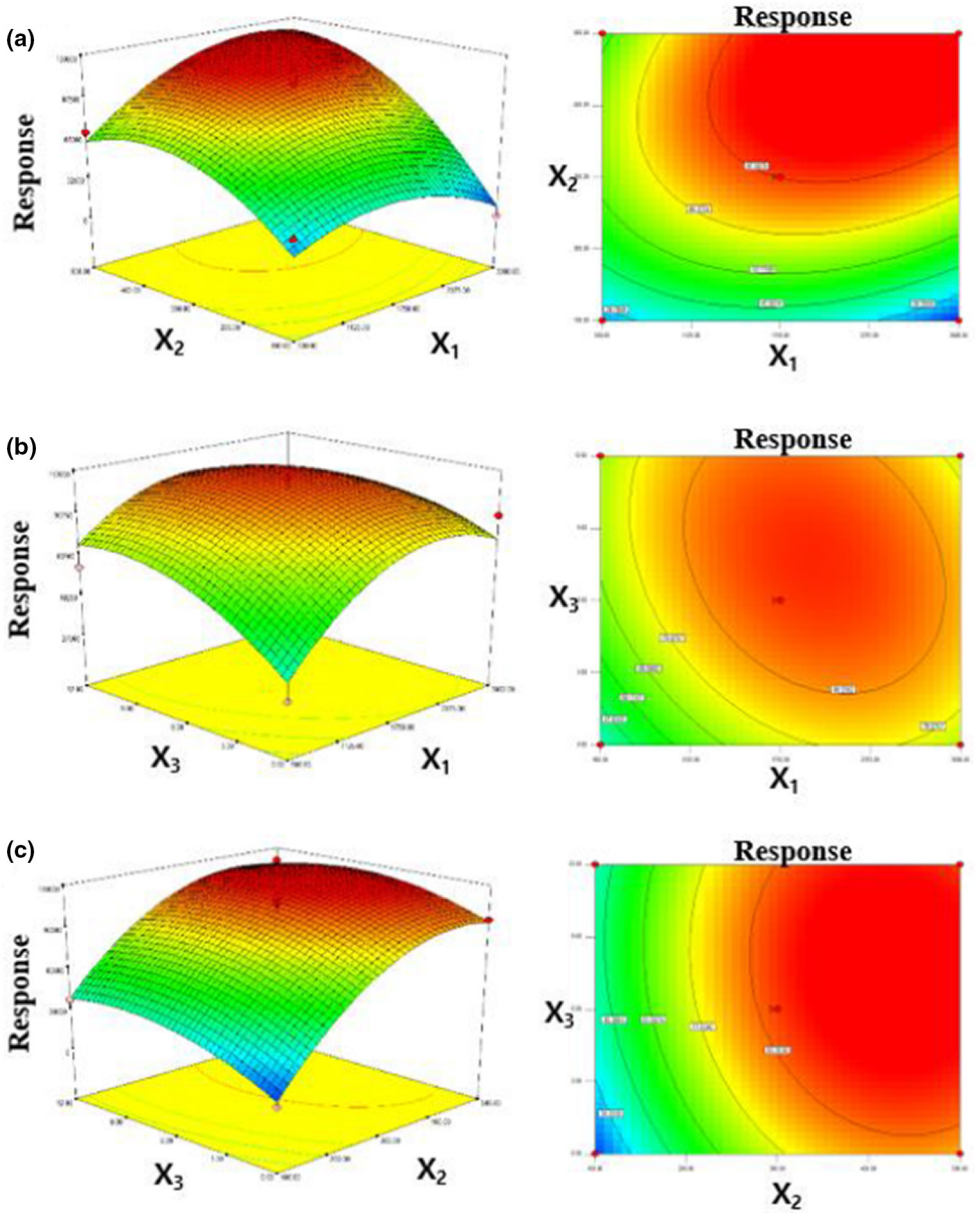
Three‐dimensional response surface plots and contour plots showing the combined effects of (a) extraction and dispersive solvent volumes, (b) extraction solvent volume and NaCl content, and (c) dispersive solvent volume and NaCl content.

The influence of the above factors on vitamin K content decreased in the order of *X*_2_ > *X*_1_ > *X*_3_ (Table 3). Equation (2) was subjected to analysis of variance to determine significance and suitability. The model response was found to be significant (*p* = .0102, *F*‐value = 10.05), and the coefficient of determination was high (*R* ^2^ = .9476). In addition, no lack‐of‐fit significance (0.1841) was observed. In general, the lack‐of‐fit value is significant if the data are not well fitted by the model and incorrect response surface values are obtained. Three‐dimensional surface plots were used to evaluate the effect of two‐parameter interactions on the response. At the center point of NaCl content (6%), vitamin K content was maximized at an extraction solvent volume of 1000–3000 μl and a dispersive solvent volume of 300–500 μl (Figure 2a). At a fixed dispersive solvent volume of 300 μl, vitamin K content was maximized at an extraction solvent volume of 1000–2000 μl and a NaCl content of 6%–9% (Figure 2b). Finally, at a fixed extraction solvent volume of 1750 μl, vitamin K content was maximized at a dispersive solvent volume of 300–500 μl and a NaCl content of 3%–12% (Figure 2c). Based on the above, the optimal conditions were chosen as extraction solvent volume = 1000 μl, dispersive solvent volume = 300 μl, and NaCl content = 6%.

**TABLE 3:** Analysis of variance data for the quadratic response surface model

Source	Sum of squares	df	Mean square	*F*‐value	*p* value	Prob. > *F*
Model	16101.59	9	1789.07	10.05	.0102	Significant
*X*_1_	840.57	1	840.57	4.72	.0818	
*X*_2_	9242.03	1	9242.03	51.94	.0008	
*X*_3_	632.61	1	632.61	3.56	.1180	
*X*_1_ *X*_2_	992.98	1	992.98	5.58	.0646	
*X*_1_ *X*_3_	243.76	1	243.76	1.37	.2946	
*X*_2_ *X*_3_	48.02	1	48.02	0.27	.6256	
*X*_1_^2^	1632.17	1	1632.17	9.17	.0291	
*X*_2_^2^	2430.38	1	2430.38	13.66	.0141	
*X*_3_^2^	589.69	1	589.69	3.31	.1283	
Residual	889.67	5	177.93			
Lack of fit	776.81	3	258.94	4.59	.1841	Not significant
Pure error	112.86	2	56.43			
Correct total	16991.26	14				

*Note*: *p* < .01, highly significant; *p* < .05, significant; *p* > .05, not significant.

Abbreviation: df, degree of freedom.

### 3.3 Method validation

**FIGURE 3:**
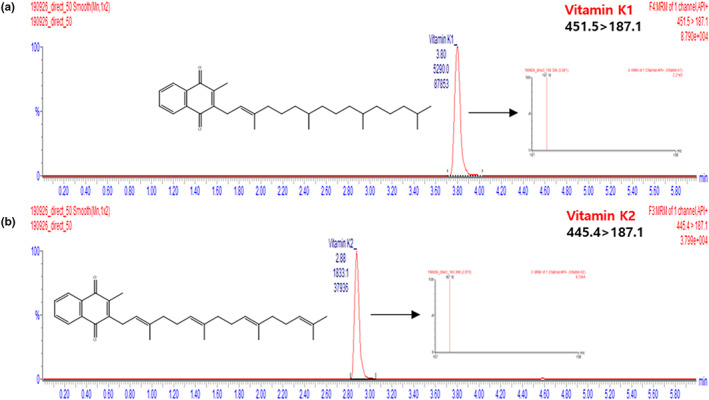
Chromatograms and mass spectra of vitamins (a) K1 and (b) K2 in vitamin‐K‐fortified samples recorded using the newly developed method.

The optimized method was validated in terms of accuracy, repeatability, LOD, and LOQ according to international guidelines (ICH, 2005). Linearity was evaluated using standard solutions of vitamin K with different concentrations (1, 5, 10, 50, 100, and 1000 μg/L), and the method was found to be highly linear (*R* ^2^ > .999). The LODs and LOQs were determined as 1.89 and 5.72 μg/L, respectively, for vitamin K1, and as 5.00 and 15.15 μg/L, respectively, for vitamin K2 (Table 4). Intra‐ and inter‐day repeatabilities were evaluated for 5, 10, and 50 μg/L standard solutions and were found to be high, with RSD values lying below 5%. The recoveries obtained for samples fortified with 5, 10, and 50 μg/L vitamin K standard solutions exceeded 90% in all cases (Table 5), which indicated that the method was sufficiently accurate for quantitative analysis. Standard additions were carried out according to EU guidelines (European Commission, 2002) by analyzing a blank sample prepared by dissolving lecithin, TW80, and olive oil in distilled water without vitamin K. Thus, the developed method was found to be well suited for the quantitation of vitamin K in fortified samples, with the representative LC‐APCI‐MS chromatograms of vitamins K1 and K2 in such samples shown in Figure 3.

**TABLE 4:** Linearity, LODs, and LOQs of the DLLME‐based method

Analyte	Regression equation^a^	Correlation coefficient (*R* ^2^)	Linear range (μg/L)	LOD (μg/L)	LOQ (μg/L)
Vitamin K1	*y* = 1773.1*x* − 23,888	.9996	1–1000	1.89	5.72
Vitamin K2	*y* = 399.4*x* − 254.45	.9999	1–1000	5.00	15.15

^a^ *y*, Analyte peak area (mAU); *x*, Analyte concentration (unit).

**TABLE 5:** Validation of DLLME‐based method

Sample	Analyte	Precision RSD (%)				Accuracy	
		Concentration (μg/L)	Intra‐day (*n* = 3)	Inter‐day (*n* = 3)	Repeatability RSD (%) (*n* = 5)	Recovery (%)	RSD (%)
NEs	Vitamin K1	5	3.83	4.38	3.67	99.76 ± 2.05	2.2
		10	3.96	3.04		101.95 ± 1.28	1.4
		50	1.17	1.16		100.48 ± 0.92	1.0
	Vitamin K2	5	2.71	2.82	3.58	99.45 ± 0.43	0.5
		10	2.33	2.38		100.19 ± 1.31	1.4
		50	3.72	3.74		101.05 ± 0.76	0.8
SLNs	Vitamin K1	5	3.83	4.38	3.67	101.03 ± 0.94	1.3
		10	3.96	3.04		103.00 ± 0.67	0.9
		50	1.17	1.16		103.44 ± 3.01	4.0
	Vitamin K2	5	2.71	2.82	3.58	100.69 ± 1.77	2.5
		10	2.33	2.38		104.04 ± 2.62	3.6
		50	3.72	3.74		99.27 ± 0.80	1.1

### 3.4 Analysis of vitamin‐fortified samples and performance comparison with Korean Food Code method

The developed method was used to quantitate vitamins K1 and K2 in two types of vitamin‐fortified samples (NEs and SLNs) using the procedure described above. All experiments were performed in triplicate, with the results listed in Table 6. The mean recoveries of vitamins K1 and K2 equaled 90.73% and 91.87%, respectively, for NE samples, whereas lower values of 73.64% and 70.99%, respectively, were obtained for SLN samples. Therefore, the developed method was found to be suitable for vitamin K quantitation in NE samples. In addition, our technique was compared with the Korean Food Code (KFDA, 2019) method. In the latter method, the fat in the analyzed sample is enzymatically decomposed and precipitated as fatty acids, and vitamin K is extracted with hexane and analyzed with a fluorescence detector using a reverse‐phase column and post‐column. The recovery of the Food Code method was less than 50% owing to sample matrix effects, while the recovery of our method exceeded 90%.

**TABLE 6:** Results of analyzing vitamin‐fortified samples using DLLME‐based and Korean Food Code methods

Sample	Analyte	DLLME‐LC‐APCI‐MS/MS		Korean Food Code	
		Recovery (%)	RSD (%)	Recovery (%)	RSD (%)
NEs	Vitamin K1	91.87 ± 1.76	4.4	46.47 ± 0.31	0.66
	Vitamin K2	90.73 ± 0.39	0.4	No analytical method^a^	
SLNs	Vitamin K1	73.64 ± 1.02	1.6	49.00 ± 1.39	4.78
	Vitamin K2	70.99 ± 0.47	0.7	No analytical method	

^a^ The Korean Food Code (KFDA, 2019) provides no analytical method for vitamin K2.

## 4 CONCLUSION

DLLME coupled with LC‐APCI‐MS/MS was successfully used to quantitate vitamin K in correspondingly loaded lipid nanocarriers. The developed method exhibited good linearity, low limits of detection and quantitation, satisfactory repeatability, and acceptable recoveries while offering the advantages of convenience, cost‐effectiveness, high sensitivity, low consumption of organic solvents, and short extraction times. Thus, our method was concluded to be sufficiently powerful, reliable, and suited for quality control purposes to present a viable alternative to the current Korean Food Code method.

## CONFLICT OF INTEREST

The authors declare that they have no conflict of interest.

## ETHICAL APROVAL

This study does not involve any human or animal testing.

## Data Availability

The data that support the findings of this study are available from the corresponding author upon reasonable request.
